# Comparison of neonatal intensive care: Trento area versus Vermont Oxford Network

**DOI:** 10.1186/1824-7288-35-5

**Published:** 2009-03-14

**Authors:** Giuseppe De Nisi, Mariarosaria Berti, Riccardo Malossi, Fabio Pederzini, Anna Pedrotti, Alberta Valente

**Affiliations:** 1 Neonatology and neonatal intensive care, S. Chiara Hospital, Trento, Italy

## Abstract

**Background:**

S. Chiara hospital is the only neonatal intensive care unit (NICU) in the Province of Trento (Italy). It serves a population of about 460000 people with about 5000 infants per year, admitting the totality of the inborn and outborn VLBWI of the province. The aim of this work is to compare mortality, morbidity and neonatal treatment of the very low birth weight infants (VLBWI) of Trento area with those recorded in the Vermont Oxford Network (VON) during 2004.

**Methods:**

In this retrospective analysis, the rates of complications and related treatments reported in VLBWI admitted in the S. Chiara NICU during the period 2000–2005 were compared with those recorded in the VON in 2004. The analysis included both the total populations and different weight groups.

**Results:**

The frequency of inborn infants was significantly higher in Trento than in VON: 91% vs 84% (MH 8.56; *p*-value 0.003). The administration of prenatal steroids (82% vs 74%; MH 7.47 and *p*-value 0.006) and caesarean section were significantly more frequent in the Trento area than in VON. In Trento significantly more VLBWI with BW ≤ 1000 grams were given surfactant prophylaxis compared with VON and significantly fewer VLBWI in every Trento weight group developed RDS (MH 18.55; *p*-value 0.00001). Overall rates of complications (CLD, PDA, NEC, IVH) were significantly lower than in the Vermont Oxford Network. In CLD and PDA the differences were marked also in infants weighting less than 1000 grams. Overall rates of PNX, PVL, severe grade of ROP and mortality were similar in the two populations. In Trento, significantly more infants were discharged on human milk than in VON, in both the overall population and in BW sub-groups.

**Conclusion:**

On the basis of this analysis, a less aggressive therapeutic strategy based on perinatal prevention in global management, such as that employed in Trento area, may be associated with an improvement in clinical outcomes in very low birth weight infants.

## Background

Very low birth weight infants (VLBWI), i.e. those weighing < 1500 grams, account for a very small percentage of all live births but make a disproportionately large contribution to neonatal mortality and morbidity [1]. In 2002, 53.9% of infants who died in the USA were VLBWI [1]. In Italy, the incidence of VLBWI is about one out of 100 live births (5000 VLBWI per year) [1,2]. Moreover, mortality rates in VLBWI are 100-fold higher than in other infants [1]. Management of VLBWI at the intensive care level is crucial for reducing mortality, morbidity and the risk of long-term complications [3,4]. In the last 20 years, the increased use of prenatal steroids and supplementary surfactant, and wider use of ventilation techniques, has provided major improvements in intensive care of VLBWI [5]. However, only a small number of studies involving large populations of VLBWI are available to date. More such studies may provide information on mortality and treatment approaches, and form the basis for more effective and less expensive therapeutic strategies [6].

VLBWI vary substantially in terms of mortality and response to therapy, according to birth weight (BW) and to gestational age (GA) [7]. Moreover, clinical outcomes may vary between different areas as a result of specific treatment strategies employed in individual intensive care units, including emergency transport [6]. Therefore, databases collecting information about VLBWI, classified on the basis of GA and BW, may be important repositories for clinical decision-making. The most notable example of such a database is that of the Vermont Oxford Network (VON) [8,9]. At its foundation in 1990, VON included 36 intensive care units. Currently, VON comprises more than 500 units worldwide, and records mortality, morbidity and treatments for about 40000 VLBWI per year. At the end of each year, the performance for each individual unit is compared with the overall VON results. In Italy, only five intensive care units were included in VON in 2004, and thus little information on VLBWI morbidity and treatments is available [1]. Therefore, therapeutic strategies based on VON data rely upon the experiences of different geographic areas [1]. Such choices may not be completely suitable for individual local units.

S. Chiara hospital is the only neonatal intensive care unit (NICU) in the Province of Trento (Italy). It serves a population of about 460000 people with about 5000 infants per year, of which one in 100 are VLBWI, admitting the totality of the inborn and outborn VLBWI of the province. Its provincial organization is based on the "in-uterus" transport to S. Chiara and emergency transport from referral delivery points for only few cases every year. S. Chiara is a 20-bed unit with eight beds dedicated to intensive care, each providing assisted ventilation and monitoring of vital parameters. NO treatment, radiography, echography and haemogas-analysis devices, and infusion pumps are also available. The working team includes 9 neonatologists and 25 nurses. Operative, surgical, orthopaedic and neuropsychiatric units are present in the paediatric department of S. Chiara. A specialised ophthalmologist is dedicated to diagnosis of retinopathy of premature (ROP). In the Trento area all the patients go directly home from S. Chiara NICU. There is no other centre where they can be transferred to. Even the follow up for the first 2 years of life is made exclusively by S. Chiara NICU of Trento. The S. Chiara neonatal intensive care unit provides emergency transport for the entire Trento area.

Rather than a comparison among NICUs, this study wants to be a comparison between two areas, the small Trento-area and the big VON-area. The aim of this retrospective analysis is to compare mortality, morbidity and treatment of VLBWI in the S. Chiara intensive care unit during 2000–2005 with those recorded in VON during 2004. In particular, this analysis focuses on infants weighing ≤ 1000 grams.

## Methods

In the Trento area, only 50 VLBWI are recorded each year compared with about 40000 in VON. Therefore, consecutive VLBWI data reported in S. Chiara during a six-year period (2000–2005) were compared with a single year for VON (2004).

Baseline data (inborn infants, use of prenatal steroids, multiple births, incidence of small for gestational age infants [SGA babies, defined for birth weight below the 10th percentile, in babies of the same gestational age [8]], caesarean sections, congenital anomalies, need for intubation in delivery room) were collected for the two populations. The incidence of respiratory distress syndrome (RDS), defined as radiologically confirmed clinical signs of respiratory distress [5], and of pneumothorax were evaluated, as well as the frequency of chronic lung disease (CLD), defined as the need for oxygen supplementation at 28 days and 36 weeks [1]. In terms of respiratory complications, the frequency of surfactant administration (Curosurf^®^; Chiesi Farmaceutici, Parma, Italy [200 mg/kg bolus]) was recorded. Surfactant was given as a prophylactic measure in the delivery room to all babies with GA < 29 weeks. In Trento area the policy about ELBWI (22–24 weeks of GA) is based on the active care with resuscitation since the 22^nd ^week. In general the Trento policy for all the VLBWI is based on the early prophylactic administration of surfactant in Trento in delivery room or in the first hours of life; this step is followed by the clinical evaluation that permits to continue with a minimal handling or, if strictly necessary, with high invasivity (catheters, antibiotics, ventilations, etc...). Furthermore, the need for nasal continuous positive airway pressure (NCPAP) and conventional ventilation (Newport and Cub Bear ventilators; Burke & Burke) was observed. Other complications recorded included: patent ductus arteriosus (PDA), as determined by echocardiography (treatment with indomethacin [Liometacen^®^, Chiesi Farmaceutici, 0.20 mg/kg/day i.v. for three days, administered over 20 minutes] was also recorded); necrotising enterocolitis (NEC), defined as gastrointestinal signs and symptoms associated with specific radiological and operative evidence [5]; total, grade III and IV (Papile's classification) intraventricular haemorhage (IVH), diagnosed with ultrasonography [5]; and cystic periventricular leukomalacia (PVL), defined on ultrasound as multiple small cysts located in the external angles of the lateral ventricles, fronto-parietal or occipital peri-ventricular white matter [5]. ROP was scored according to international classification [5]. ROP screening frequency and results, and the need for surgical intervention for this condition were also evaluated. Overall mortality was calculated, as well as the total number of discharged infants and those discharged on human milk.

The incidence of each parameter was calculated in both the overall population and sub-groups defined by BW (501–750 grams, 751–1000 grams, 1001–1250 grams, 1251–1500 grams). Incidences were compared using odds ratios (OR) and the respective 95% confidence intervals (95% CI). The differences between the two populations were calculated with the Mantel-Haenzel estimate (MH).

## Results

A total of 250 VLBWI in the Trento area and 38895 in VON were evaluated. Baseline data for the two populations, and BW-defined sub-groups, are summarised in Table 1 (Additional file 1).

The mean birth weight in each BW category is similar in the two populations. VON in 2004 did not report the mean gestational age.

Table 2 (Additional file 2) lists the prenatal data for the two populations. The frequency of inborn infants was significantly higher in Trento than in VON: 91% vs 84% (MH 8.56; *p*-value 0.003). In addition, some treatments, such as the administration of prenatal steroids (82% vs 74%; MH 7.47 and *p*-value 0.006) and caesarean section were significantly more frequent in the Trento area than in VON. The frequencies of congenital anomalies, multiple births were similar between Trento and VON. SGA infants were less frequent in Trento.

Table 3 (Additional file 3) lists the incidence of respiratory complications and related treatments in the two populations. In Trento the overall need for intubation was significantly less frequent in the sub-group with BW 1251–1500 grams, but significantly more VLBWI with BW ≤ 1000 grams were given surfactant prophylaxis compared with VON. Compared with VON, significantly fewer VLBWI in every Trento weight group developed RDS, and therefore needed specific treatment (MH 18.55; *p*-value 0.00001), with the use of conventional ventilation and nasal CPAP (never before mechanical ventilation and never with high flow nasal cannula) significantly less frequent in Trento. Only in the sub-group with BW 501–750 grams the frequency was similar in the two populations. Greater use of surfactant prophylaxis and lower incidence of RDS in Trento compared with VON were particularly evident in the 501–1000 grams group. The same trend was found for CLD at 36 weeks: 5% vs 36% in VON (MH 102.5; p-value 0.00000). In Trento postnatal steroids for CLD are not used. The overall frequency of pneumothorax was similar in the two populations.

Table 4 (Additional file 4) summarises the incidence of other complications and of indomethacin administration. In Trento, PDA (14% vs 37%; MH 54.51; *p*-value 0.00000) and subsequent use of indomethacin (12% vs 34%; MH 55.65; *p*-value 0.00000) were significantly less frequent than in VON. This difference was greatest in the two lowest BW groups, as was also the case with respiratory complications. In Trento, NEC (2% vs 6%; MH 8.57; *p*-value 0.003) was significantly less frequent than in VON. Moreover, even if all the IVHs were significantly less frequent in Trento than in VON, the analysis of IVH-grade did not show any difference. There were no significant differences in the incidence of PVL.

Table 5 (Additional file 5) lists the frequencies of other parameters analysed, including mortality. Screening for ROP was significantly more common in Trento than in VON (83% vs 67%; MH 29.5; *p*-value 0.000001). The overall incidence of ROP was significantly lower in the S. Chiara intensive care unit, in all the BW sub-groups. No significant difference was evident in the incidence of severe grade of ROP.

**Figure 1:**
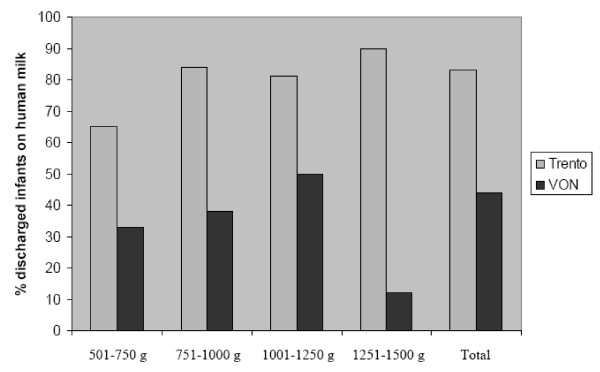
**Percentage of very low birth weight infants discharged on human milk in Trento area (2000–2005) and VON (2004)**.

Overall mortality was comparable in the two populations (10% and 14% in Trento and VON, respectively). Furthermore, in Trento, significantly more infants were discharged on human milk than in VON, in both the overall population (MH 139.7; *p*-value 0.00000) and in BW sub-groups [Figure 1].

## Discussion

This analysis reports the complications and treatments in the S. Chiara hospital (Trento, Italy) neonatal intensive care unit during 2000–2005. As the only intensive care centre in Trento (an area comprising 6200 km^2^), and serving the entire population (500000 inhabitants, with 5000 newborns per year), S. Chiara data might be an important source of epidemiological information. Data recorded in S. Chiara were compared with the entire VON database (S. Chiara is part of this network), therefore comparing a small area (2000–2005) with a global group, represented by VON (2004). In this way, it is possible to critically evaluate therapeutic strategies used in S. Chiara, in a global management of a perinatal area.

The two populations had similar rates of congenital anomalies, SGA infants, and multiple births; some intervention strategies were significantly more common in Trento than in VON. For instance, caesarean section, which is a common choice in Italy, especially in recent years [10].

In Trento, the use of prenatal steroids and the use of supplementary surfactant prophylaxis is significantly more common than in VON. The surfactant strategy is based on a multicentre retrospective European study that suggested that prophylactic surfactant administered within 15 minutes of delivery is associated with a significantly greater reduction in RDS than rescue therapy (administration after 15 minutes of life) [11]. Moreover, a head-to-head comparison of surfactant prophylaxis versus rescue therapy confirmed the same finding, suggesting that prophylaxis is associated with a reduction in mortality [12].

The use of prenatal steroids with the greater use of surfactant prophylaxis may be associated with the lower incidence of RDS reported in Trento. Effective management of this condition could reduce the risk of CLD, as suggested by the present analysis and by another study [13]. Surfactant prophylaxis, administered within the first few minutes of birth in the delivery room, may also result in fewer infants requiring assisted ventilation, thus reducing invasive procedures without worsening clinical outcomes [11]. The reduction in frequency of respiratory complications, as CLD, in Trento compared with VON is particularly evident in the two lowest BW groups (501–750 grams and 751–1000 grams), which are characterised by a particularly high risk. In these groups, surfactant prophylaxis was more common, further suggesting that this treatment may be associated with important improvements in clinical outcomes and better management of neonatal respiration, in terms of peak inspiration volume, intermittent positive pressure ventilation and fraction of inhaled oxygen. These results are consistent with the multicentre study by Bevilacqua *et al*, in which surfactant prophylaxis was associated with a lower incidence of RDS compared with rescue therapy [11]. It is noteworthy that VLBWI receiving surfactant prophylaxis had a lower BW than those treated with rescue therapy [11].

In Trento, the overall rates of PDA, NEC and IVH were lower than in VON, occasionally reaching statistical significance. As observed for respiratory complications, the difference was greatest in the lowest BW groups. Overall, these data are comparable with those reported in the Bevilacqua study [11]. It may be that RDS is less common in Trento area because of several factors: more prenatal steroids; surfactant in delivery room; low amount of infused liquids, thanks to the fact that in Trento the enteral feeding with bank human milk and mother milk is preferred. This management, mainly in the first week of life, gives as a consequence high weight loss (about 20%), less PDA, less RDS and less CLD, but also less indomethacin treatment, less catheters and less antibiotics (with lower risk of complications). In Trento it is usually used only one dose of surfactant (in delivery room or in NICU) with extubation as soon as possible, also in delivery room. It is preferred NCPAP instead of mechanical ventilation when the baby permits it.

Even the lower risk of NEC in Trento compared with VON may be associated with specific therapeutic strategies of the S. Chiara intensive care unit, such as the very early administration of human milk (from the second hour from birth, when possible). In fact, the percentage of discharged infants receiving human milk was significantly higher in Trento than in VON (83% vs. 44%, respectively). Since 1993, the S. Chiara unit has employed the early exclusive enteral feeding (EEEF) protocol that is widely used in Scandinavian countries [14]. This protocol, which is targeted to specific VLBWI conditions, is based on the exclusive administration of human milk, either from the mother or from a donor, to VLBWI weighing 750–1250 grams and with GA > 26 weeks. The EEEF protocol may be suitable for VLBWI undergoing CPAP, but cannot be suggested for those treated with mechanical ventilation or presenting with asphyxia, metabolic acidosis, hypotension, sepsis or persistent hypoglycaemia. During 2000–2005 in S. Chiara, the EEEF protocol was initiated in 51.4% of VLBWI weighing 750–1250 grams and with a GA > 26 weeks. Among these infants, only one out of five (10.3% of the overall population) required further nutritional support, while the majority (41.0% of the total) did not.

Several studies have confirmed the importance of VLBWI feeding with human milk. The high content of oligosaccharides in human milk may improve the development of immune system and prevent onset of NEC [15]. The administration of human milk to VLBWI is recommended by *American Academy of Pediatrics *and *Canadian Pediatrics Society *because of its excellent energetic properties and for its important effects on neural, cognitive and psychological development [16,17]. In particular, feeding with human milk is of great importance in VLBWI weighing less than 1000 grams, because it is associated with significant improvements in survival and clinical outcomes [18]. In Trento, significantly more VLBWI in both the overall population (MH 139.7; *p*-value 0.00000) and in BW sub-groups were discharged on human milk, compared with VON.

Screening for ROP was about 2.5 times more common in Trento than in VON. This strategy allows more rapid diagnosis of the potential presence of ROP. The frequency of this condition (overall and severe grades) was lower in Trento than in VON. An early diagnosis of ROP is of particular importance to prevent the risk of short- and long-term damage to vision [19].

Mortality was comparable in the two populations, although a trend towards lower mortality in Trento was evident, especially in infants with BW ≤ 750 grams. It is possible that the therapeutic strategies adopted in Trento, such as surfactant prophylaxis and widespread use of human milk, may be associated with a reduction in complications and, as a consequence, with a lower mortality in this high-risk class of VLBWI.

## Conclusion

The comparison between VLBWI data collected during 2000–2005 in the S. Chiara intensive care unit of Trento and those derived from the VON database shows that the frequency of the most important complications associated with intensive treatment was, in most cases, lower in the Trento area. This difference may be due, at least in part, to a greater use of prenatal steroids, to a less aggressive surfactant approach, based on prophylactic treatment instead of rescue therapy, and greater feeding with human milk using the EEEF protocol, which may be associated with better development and lower incidence of NEC and other complications. It is noteworthy that surfactant prophylaxis is associated with reduced need for ventilation assistance. Other factors, such as more extensive screening for ROP, might contribute to decrease time and cost associated with intensive care and to limit short- and long-term consequences. These differences are particularly evident in lower BW infants, which present a higher risk.

In conclusion, this study suggests that a less aggressive therapeutic strategy, mostly based on prevention and on global management, may be associated with an improvement in clinical outcomes in preterm infants.

## Competing interests

The authors declare that they have no competing interests.

## Authors' contributions

GDN had primary responsibility for protocol development, patients enrollment, data analysis and writing the text. MB and RM were responsible for patients evaluation schedule and Oxford Data Base maintenance. FP, AP, and AV contributed to the protocol development and execution of the study.

## Supplementary Material

Additional file 1 **Table 1. **Baseline data for the Trento and VON populations. Click here for file

Additional file 2 **Table 2.** Prenatal data for the Trento and VON populations. Click here for file

Additional file 3 **Table 3.** Incidence of respiratory complications and related treatments in Trento and VON. Click here for file

Additional file 4 **Table 4. **Incidence of non-respiratory complications and indomethacin administration in Trento and VON. Click here for file

Additional file 5 **Table 5.** Incidence of ROP and mortality in Trento and VON. Click here for file
