# Supplementary material for: Comparison of neonatal intensive care: Trento area versus Vermont Oxford Network
Source: Ital J Pediatr. 2009 Mar 14;35:5. doi: 10.1186/1824-7288-35-5 (PMC2687545; doi:10.1186/1824-7288-35-5)
Supplement: Additional file 1 — Table 1. Baseline data for the Trento and VON populations. [file 1824-7288-35-5-S1.pdf]

Tab. 1. Baseline data for the Trento and VON populations.

	501-750 g		751-1000 g		1001-1250 g		1251-1500 g		All 501-1500 g	
	**Trento**	**VON**	**Trento**	**VON**	**Trento**	**VON**	**Trento**	**VON**	**Trento**	**VON**
**Number of cases**	34	7614	50	8943	80	10003	86	12335	250	38895
**Birth weight (mean ± SD)**	649 ± 53	637 ± 58	861 ± 64	872 ± 130	1139 ± 69	1135 ± 93	1387 ± 61	1373 ± 196	1081 ± 241	1053 ± 261
**Gestational age (mean ± SD)**	25 ± 1.4	*	27 ± 1.8	*	29 ± 2.0	*	31 ± 1.6	*	28 ± 2.9	*
**Male (%)**	47	50	28	52	49	51	45	51	43	51

** not found*
