# Supplementary material for: Comparison of neonatal intensive care: Trento area versus Vermont Oxford Network
Source: Ital J Pediatr. 2009 Mar 14;35:5. doi: 10.1186/1824-7288-35-5 (PMC2687545; doi:10.1186/1824-7288-35-5)
Supplement: Additional file 2 — Table 2. Prenatal data for the Trento and VON populations. [file 1824-7288-35-5-S2.pdf]

**Tab. 2.** Prenatal data for the Trento and VON populations.

| | 501-750 g | | | | 751-1000 g | | | | 1001-1250 g | | | | 1251-1500 g | | | | All 501-1500 g | | | |
|---|---|---|---|---|---|---|---|---|---|---|---|---|---|---|---|---|---|---|---|---|
| | **Trento** | **VON** | **OR (95% CI)** | **MH (*p*-value)** | **Trento** | **VON** | **OR (95% CI)** | **MH (*p*-value)** | **Trento** | **VON** | **OR (95% CI)** | **MH (*p*-value)** | **Trento** | **VON** | **OR (95% CI)** | **MH (*p*-value)** | **Trento** | **VON** | **OR (95% CI)** | **MH (*p*-value)** |
| **Number of cases** | 34 | 7614 | | | 50 | 8943 | | | 80 | 10003 | | | 86 | 12335 | | | 250 | 38895 | | |
| **Inborn** | 33 (97%) | 6320 (83%) | 6.76 (0.99-133.) | 4.75 (0.03) | 44 (88%) | 7244 (81%) | 1.72 (070-4.49) | 1.58 (0.21) | 71 (89%) | 8403 (84%) | 1.50 (0.72-3.22) | 1.33 (0.25) | 79 (92%) | 10485 (85%) | 1.99 (0.89-4.71) | 3.16 (0.08) | 227 (91%) | 32672 (84%) | 1.88 (1.20-2.96) | 8.56 (0.003) |
| **Prenatal steroids** | 26 (76%) | 5101 (67%) | 1.60 (0.69-3.84) | 1.38 (0.24) | 39 (78%) | 6797 (76%) | 1.12 (0.55-2.32) | 0.11 (0.74) | 70 (88%) | 7802 (78%) | 1.97 (0.98-4.08) | 4.19 (0.04) | 69 (80%) | 9005 (73%) | 1.50 (0.86-2.65) | 2.27 (0.13) | 204 (82%) | 28782 (74%) | 1.56 (1.12-2.18) | 7.47 (0.006) |
| **Cesarean section** | 24 (71%) | 4492 (59%) | 1.67 (0.76-3.73) | 1.88 (0.17) | 42 (84%) | 6260 (70%) | 2.25 (1.01-5.19) | 4.65 (0.03) | 69 (86%) | 7202 (72%) | 2.44 (1.25-4.88) | 8.01 (0.005) | 78 (91%) | 8511 (69%) | 4.38 (2.04-9.79) | 18.85 (0.00001) | 213 (85%) | 26449 (68%) | 2.71 (1.89-3.91) | 33.83 (0.0000) |
| **Multiple births** | 6 (18%) | 1904 (25%) | 0.64 (0.24-1.63) | 0.98 (0.32) | 10 (20%) | 2325 (26%) | 0.71 (0.33-1.48) | 0.93 (0.33) | 13 (16%) | 2901 (29%) | 0.48 (0.25-0.89) | 6.28 (0.01) | 25 (29%) | 4071 (33%) | 0.83 (0.51-1.36) | 0.60 (0.44) | 54 (22%) | 11280 (29%) | 0.67 (0.49-0.92) | 6.61 (0.01) |
| **Congenital anomalies** | 1 (3%) | 305 (4%) | 0.73 (---) | 0.10 (0.75) | 2 (4%) | 447 (5%) | 0.79 (0.13-3.33) | 0.10 (0.75) | 5 (6%) | 500 (5%) | 1.27 (0.45-3.27) | 0.26 (0.61) | 7 (8%) | 617 (5%) | 1.68 (0.71-3.79) | 1.76 (0.18) | 15 (6%) | 1945 (5%) | 1.21 (0.69-2.09) | 0.52 (0.47) |
| **Small for gestational age** | 3 (9%) | 1218 (16%) | 0.51 (0.12-1.74) | 1.30 (0.25) | 4 (8%) | 1252 (14%) | 0.53 (0.16-1.55) | 1.49 (0.22) | 7 (9%) | 2001 (20%) | 0.38 (0.16-0.86) | 6.30 (0.01) | 14 (16%) | 3207 (26%) | 0.55 (0.30-1.01) | 4.20 (0.04) | 28 (11%) | 7779 (20%) | 0.50 (0.33-0.76) | 12.05 (0.0005) |

*Data are shown as number of cases and( %)*
*OR: odds ratio; 95% CI: 95% confidence interval; MH: Mantel-Haenszel estimate*
