# Supplementary material for: Comparison of neonatal intensive care: Trento area versus Vermont Oxford Network
Source: Ital J Pediatr. 2009 Mar 14;35:5. doi: 10.1186/1824-7288-35-5 (PMC2687545; doi:10.1186/1824-7288-35-5)
Supplement: Additional file 3 — Table 3. Incidence of respiratory complications and related treatments in Trento and VON. [file 1824-7288-35-5-S3.pdf]

Tab. 3. Incidence of respiratory complications and related treatments in Trento and VON.

	501-750 g			751-1000 g			1001-1250 g			1251-1500 g			All 501-1500 g		
	**Trento**	**VON**	**OR (95% CI)**<br>**MH (*p*-value)**	**Trento**	**VON**	**OR (95% CI)**<br>**MH (*p*-value)**	**Trento**	**VON**	**OR (95% CI)**<br>**MH (*p*-value)**	**Trento**	**VON**	**OR (95% CI)**<br>**MH (*p*-value)**	**Trento**	**VON**	**OR (95% CI)**<br>**MH (*p*-value)**
**Number of cases**	34	7614		50	8943		80	10003		86	12335		250	38895	
**Intubation in delivery room**	28 (82%)	6167 (81%)	1.09 (0.43-2.95)<br>0.04 (0.84)	36 (72%)	6618 (74%)	0.90 (0.47-1.76)<br>0.10 (0.75)	37 (46%)	4901 (49%)	0.90 (0.56-1.42)<br>0.24 (0.62)	12 (14%)	3454 (28%)	0.42 (0.22-0.79)<br>8.38 (0.004)	113 (45%)	21003 (54%)	0.70 (0.54-0.91)<br>7.74 (0.005)
**Surfactant prophylaxis**	28 (82%)	3655 (48%)	5.05 (1.99-13.6)<br>15.99 (0.00006)	33 (66%)	3935 (44%)	2.47 (1.33-4.63)<br>9.76 (0.002)	28 (35%)	2701 (27%)	1.46 (0.89-2.36)<br>2.57 (0.11)	6 (7%)	1604 (13%)	0.50 (0.20-1.20)<br>2.75 (0.097)	95 (38%)	11669 (30%)	1.43 (1.10-1.86)<br>7.56 (0.006)
**RDS**	31 (91%)	7157 (94%)	0.66 (0.19-2.71)<br>0.48 (0.49)	38 (76%)	7780 (87%)	0.47 (0.24-0.96)<br>5.29 (0.02)	51 (64%)	7202 (72%)	0.68 (0.42-1.11)<br>2.67 (0.10)	35 (41%)	6661 (54%)	0.58 (0.37-0.92)<br>6.08 (0.014)	155 (62%)	28782 (74%)	0.57 (0.44-0.75)<br>18.55 (0.00001)
**Pneumothorax**	2 (6%)	761 (10%	0.56 (0.09-2.41)<br>0.64 (0.42)	1 (2%)	537 (6%)	0.32 (0.02-2.14)<br>1.42 (0.23)	5 (6%)	300 (3%)	2.16 (0.76-5.59)<br>2.86 (0.09)	2 (2%)	370 (3%)	0.77 (0.13-3.19)<br>0.13 (0.71)	10 (4%)	1945 (5%)	0.79 (0.40-1.53)<br>0.52 (0.47)
**NCPAP**	20 (59%)	4492 (59%)	0.99 (0.48-2.07)<br>0.00 (0.98)	32 (64%)	6797 (76%)	0.56 (0.30-1.04)<br>3.92 (0.05)	50 (63%)	7102 (71%)	0.68 (0.42-1.10)<br>2.78 (0.095)	38 (44%)	7154 (58%)	0.57 (0.37-0.90)<br>6.68 (0.01)	140 (56%)	25671 (66%)	0.66 (0.51-0.85)<br>11.06 (0.0009)
**Conventional ventilation**	30 (88%)	7005 (92%)	0.65 (0.22-2.19)<br>0.65 (0.42)	36 (72%)	7602 (85%)	0.45 (0.24-0.88)<br>6.57 (0.01)	39 (49%)	6602 (66%)	0.49 (0.31-0.78)<br>10.50 (0.001)	21 (24%)	5551 (45%)	0.39 (0.23-0.66)<br>14.63 (0.0001)	126 (50%)	26449 (68%)	0.48 (0.37-0.62)<br>35.30 (0.00000)
**CLD after 28 days**	10 (29%)	7005 (92%)	0.04 (0.02-0.08)<br>174.65 (0.0000)	10 (20%)	6618 (74%)	0.09 (0.04-0.18)<br>74.80 (0.00000)	11 (14%)	4301 (43%)	0.21 (0.11-0.41)<br>27.73 (0.00000)	2 (2%)	2714 (22%)	0.08 (0.01-0.35)<br>19.35 (0.00001)	33 (13%)	20614 (53%)	0.13 (0.09-0.20)<br>157.8 (0.00000)
**CLD after 36 weeks**	5 (15%)	5330 (70%)	0.07 (0.03-0.20)<br>49.05 (0.00000)	6 (12%)	4382 (49%)	0.14 (0.05-0.35)<br>27.24 (0.00000)	2 (3%)	2701 (27%)	0.07 (0.01-0.29)<br>24.28 (0.00000)	0 (0%)	1850 (15%)	0.00 (0.00-0.32)<br>15.15 (0.0001)	13 (5%)	14002 (36%)	0.10 (0.05-0.17)<br>102.5 (0.00000)

Data are shown as number of cases and(%)
OR: odds ratio; 95% CI: 95% confidence interval; MH: Mantel-Haenszel estimate
