# Supplementary material for: Comparison of neonatal intensive care: Trento area versus Vermont Oxford Network
Source: Ital J Pediatr. 2009 Mar 14;35:5. doi: 10.1186/1824-7288-35-5 (PMC2687545; doi:10.1186/1824-7288-35-5)
Supplement: Additional file 4 — Table 4. Incidence of non-respiratory complications and indomethacin administration in Trento and VON. [file 1824-7288-35-5-S4.pdf]

Tab. 4. Incidence of non-respiratory complications and indomethacin administration in Trento and VON.

	501-750 g			751-1000 g			1001-1250 g			1251-1500 g			All 501-1500 g		
	**Trento**	**VON**	**OR (95% CI)**<br>**MH (*p*-value)**	**Trento**	**VON**	**OR (95% CI)**<br>**MH (*p*-value)**	**Trento**	**VON**	**OR (95% CI)**<br>**MH (*p*-value)**	**Trento**	**VON**	**OR (95% CI)**<br>**MH (*p*-value)**	**Trento**	**VON**	**OR (95% CI)**<br>**MH (*p*-value)**
**Number of cases**	34	7614		50	8943		80	10003		86	12335		250	38895	
**PDA**	8<br>(24%)	4340<br>(57%)	0.23 (0.10-0.64)<br>15.46 (0.00008)	12<br>(24%)	4561<br>(51%)	0.30 (0.15-0.60)<br>14.50 (0.0001)	11<br>(14%)	3301<br>(33%)	0.32 (0.16-0.63)<br>13.33 (0.0003)	5<br>(6%)	2344<br>(19%)	0.26 (0.09-0.67)<br>9.69 (0.002)	36<br>(14%)	14391<br>(37%)	0.29 (0.20-0.41)<br>54.51 (0.00000)
**Indomethacin**	8<br>(24%)	4188<br>(55%)	0.25 (0.10-0.58)<br>13.54 (0.0002)	11<br>(22%)	4472<br>(50%)	0.28 (0.14-0.57)<br>15.60 (0.00007)	8<br>(10%)	3001<br>(30%)	0.26 (0.12-0.56)<br>15.16 (0.0001)	2<br>(2%)	1727<br>(14%)	0.15 (0.02-0.60)<br>9.71 (0.002)	29<br>(12%)	13224<br>(34%)	0.25 (0.17-0.38)<br>55.65 (0.00000)
**NEC**	1<br>(3%)	761<br>(10%)	0.27 (0.01-1.86)<br>1.88 (0.17)	2<br>(4%)	715<br>(8%)	0.48 (0.08-2.01)<br>1.08 (0.30)	1<br>(1%)	500<br>(5%)	0.24 (0.01-1.60)<br>2.36 (0.12)	0<br>(0%)	370<br>(3%)	0.00 (0.00-1.82)<br>2.66 (0.10)	4<br>(2%)	2334<br>(6%)	0.25 (0.08-0.71)<br>8.57 (0.003)
**IVH**	12<br>(35%)	3502<br>(46%)	0.64 (0.30-1.35)<br>1.56 (0.21)	16<br>(32%)	2862<br>(32%)	1.00 (0.53-1.88)<br>0.00 (0.10)	7<br>(9%)	2101<br>(21%)	0.36 (0.15-0.81)<br>7.21 (0.007)	7<br>(8%)	1974<br>(16%)	0.47 (0.20-1.05)<br>3.94 (0.05)	42<br>(17%)	10113<br>(26%)	0.57 (0.41-0.81)<br>10.95 (0.0009)
**I grade**	2<br>(6%)	914<br>(12%)	0.46 (0.08-1.96)<br>1.20 (0.27)	6<br>(12%)	1073<br>(12%)	1.00 (0.38-2.45)<br>0.00 (0.10)	3<br>(4%)	1100<br>(11%)	0.32 (0.08-1.04)<br>4.28 (0.04)	4<br>(5%)	1234<br>(10%)	0.44 (0.14-1.24)<br>2.73 (0.98)	15<br>(6%)	4278<br>(11%)	0.52 (0.29-0.89)<br>6.36 (0.012)
**II grade**	2<br>(6%)	838<br>(11%)	0.51 (0.08-2.16)<br>0.91 (0.34)	3<br>(6%)	626<br>(7%)	0.85 (0.21-2.84)<br>0.08 (0.78)	1<br>(1%)	500<br>(5%)	0.24 (0.01-1.60)<br>2.36 (0.12)	1<br>(1%)	370<br>(3%)	0.38 (0.02-2.52)<br>0.99 (0.32)	7<br>(3%)	2334<br>(6%)	0.45 (0.20-0.99)<br>4.53 (0.03)
**III grade**	5<br>(15%)	685<br>(9%)	1.74 (0.59-4.74)<br>1.34 (0.25)	2<br>(4%)	537<br>(6%)	0.65 (0.11-2.74)<br>0.35 (0.55)	2<br>(3%)	300<br>(3%)	0.83 (0.0-3.45)<br>0.07 (0.79)	0<br>(0%)	247<br>(2%)	0.00 (0.00-2.77)<br>1.76 (0.18)	9<br>(4%)	1556<br>(4%)	0.90 (0.43-1.80)<br>0.10 (0.75)
**IV grade**	3<br>(9%)	1066<br>(14%)	0.59 (0.14-2.03)<br>0.75 (0.38)	5<br>(10%)	626<br>(7%)	1.48 (0.51-3.90)<br>0.69 (0.41)	1<br>(1%)	200<br>(2%)	0.62 (0.03-4.14)<br>0.23 (0.63)	2<br>(2%)	123<br>(1%)	2.36 (0.0-9.90)<br>1.51 (0.22)	11<br>(4%)	1945<br>(5%)	0.87 (0.45-1.64)<br>0.19 (0.66)
**Cystic PVL**	1<br>(3%)	381<br>(5%)	0.58 (0.03-3.92)<br>0.30 (0.58)	1<br>(2%)	358<br>(4%)	0.49 (0.03-3.29)<br>0.52 (0.47)	2<br>(3%)	300<br>(3%)	0.83 (0.0-3.45)<br>0.07 (0.79)	2<br>(2%)	247<br>(2%)	1.17 (0.0-4.84)<br>0.05 (0.83)	6<br>(2%)	1167<br>(3%)	0.79 (0.32-1.85)<br>0.31 (0.58)

Data are shown as number of cases and(%)
OR: odds ratio; 95% CI: 95% confidence interval; MH: Mantel-Haenszel estimate
