# Supplementary material for: Comparison of neonatal intensive care: Trento area versus Vermont Oxford Network
Source: Ital J Pediatr. 2009 Mar 14;35:5. doi: 10.1186/1824-7288-35-5 (PMC2687545; doi:10.1186/1824-7288-35-5)
Supplement: Additional file 5 — Table 5. Incidence of ROP and mortality in Trento and VON. [file 1824-7288-35-5-S5.pdf]

Tab. 5. Incidence of ROP and mortality in Trento and VON.

	501-750 g			751-1000 g			1001-1250 g			1251-1500 g			All 501-1500 g		
	**Trento**	**VON**	**OR (95% CI)**<br>**MH (*p*-value)**	**Trento**	**VON**	**OR (95% CI)**<br>**MH (*p*-value)**	**Trento**	**VON**	**OR (95% CI)**<br>**MH (*p*-value)**	**Trento**	**VON**	**OR (95% CI)**<br>**MH (*p*-value)**	**Trento**	**VON**	**OR (95% CI)**<br>**MH (*p*-value)**
**Number of cases**	34	7614		50	8943		80	10003		86	12335		250	38895	
**ROP screening**	23 (68%)	4797 (63%)	1.23 (0.57-2.68)<br>0.31 (0.58)	43 (86%)	7244 (81%)	1.44 (0.62-3.51)<br>0.81 (0.37)	72 (90%)	7502 (75%)	3.00 (1.39-6.73)<br>9.56 (0.002)	70 (81%)	6661 (54%)	3.73 (2.11-6.68)<br>25.8 (0.000004)	208 (83%)	26060 (67%)	2.44 (1.73-3.45)<br>29.5 (0.000001)
**ROP positives**	14 (61%)	3789 (79%)	0.41 (0.17-1.04)<br>4.51 (0.03)	11 (26%)	4057 (56%)	0.27 (0.13-0.56)<br>16.04 (0.00006)	3 (4%)	2101 (28%)	0.11 (0.03-0.37)<br>20.20 (0.000007)	0 (0%)	866 (13%)	0.00 (0.00-0.46)<br>10.44 (0.001)	28 (13%)	10684 (41%)	0.22 (0.15-0.34)<br>64.7 (0.000000)
**ROP severe grade (III-IV)**	5 (22%)	1487 (31%)	0.62 (0.20-1.77)<br>0.92 (0.34)	3 (7%)	869 (12%)	0.55 (0.14-1.85)<br>1.02 (0.31)	0 (0%)	225 (3%)	0.00 (0.00-2.19)<br>2.23 (0.14)	0 (0%)	67 (1%)	0.00 (0.00-6.93)<br>0.71 (0.40)	8 (4%)	2606 (10%)	0.36 (0.16-0.75)<br>8.72 (0.003)
**Need for surgical interv.**	2 (9%)	240 (5%)	1.81 (0.42-7.64)<br>0.65 (0.42)	1 (2%)	1087 (15%)	0.13 (0.01-0.91)<br>5.41 (0.02)	0 (0%)	525 (7%)	0.00 (0.00-0.90)<br>5.41 (0.02)	0 (0%)	67 (1%)	0.00 (0.00-6.93)<br>0.71 (0.40)	3 (1%)	1919 (7%)	0.18 (0.05-0.59)<br>10.67 (0.001)
**Deceased**	11 (32%)	3350 (44%)	0.61 (0.28-1.31)<br>1.86 (0.17)	7 (14%)	1252 (14%)	1.00 (0.41-2.32)<br>0.00 (0.99)	3 (4%)	600 (6%)	0.61 (0.15-2.01)<br>0.71 (0.40)	3 (3%)	493 (4%)	0.87 (0.22-2.85)<br>0.06 (0.81)	24 (10%)	5445 (14%)	0.65 (0.42-1.01)<br>4.00 (0.045)
**Discharged**	23 (68%)	4264 (56%)	1.64 (0.76-3.59)<br>1.86 (0.17)	43 (86%)	7691 (86%)	1.00 (0.43-2.44)<br>0.00 (0.99)	77 (96%)	9403 (94%)	1.64 (0.50-6.51)<br>0.71 (0.40)	83 (97%)	11842 (96%)	1.15 (0.35-4.57)<br>0.06 (0.81)	226 (90%)	33450 (86%)	1.53 (0.99-2.39)<br>4.00 (0.045)
**Discharged on human milk**	15 (65%)	1407 (33%)	3.81 (1.52-9.81)<br>10.71 (0.001)	36 (84%)	2923 (38%)	8.39 (3.57-20.6)<br>37.83 (0.00000)	62 (81%)	4701 (50%)	4.13 (2.29-7.59)<br>28.4 (0.000001)	75 (90%)	1421 (12%)	68.7 (31.9-154)<br>461 (0.000000)	188 (83%)	14718 (44%)	6.30 (4.39-9.07)<br>139.7 (0.00000)

Data are shown as number of cases and(%)

OR: odds ratio; 95% CI: 95% confidence interval; MH: Mantel-Haenszel estimate
